# NGF-Hydrogel Ameliorates Aberrant Adult Hippocampal Neurogenesis and Improves Hippocampal Remodeling After Epilepsy

**DOI:** 10.3390/cimb48060608

**Published:** 2026-06-10

**Authors:** Yuanyuan Bai, Kangzhen Chen, Taojie Yao, Shengbo Shi, Hongmei Duan, Peng Hao, Wen Zhao, Yudan Gao, Xiaoguang Li, Zhaoyang Yang

**Affiliations:** Department of Neurobiology, School of Basic Medical Sciences, Capital Medical University, Beijing 100069, China

**Keywords:** temporal lobe epilepsy, NGF-hydrogel, hippocampal neurogenesis, neural repair, dentate gyrus, hippocampal remodeling

## Abstract

Temporal lobe epilepsy (TLE) is a common drug-resistant epilepsy characterized by recurrent seizures, cognitive impairment, aberrant adult hippocampal neurogenesis, inhibitory circuit disruption, and persistent inflammatory remodeling. Current anti-seizure medications primarily offer symptomatic control and do not target the progressive structural and functional deterioration of epileptic hippocampal networks. Here, we investigated whether local nerve growth factor (NGF)-hydrogel delivery during the latent phase after status epilepticus could mitigate hippocampal pathological remodeling and improve long-term outcomes in a kainic acid (KA)-induced mouse model (utilizing C57BL/6J and *Nestin-CreERT2* mice). Animals were randomly assigned to three groups: the saline control group, the untreated KA epilepsy group, and the KA + NGF-hydrogel treatment group. NGF-hydrogel was administered into hippocampal Cornu Ammonis 1 (CA1) beginning 3 days post-kainic acid and repeated every 15 days. Histological, immunofluorescence, circuit-tracing, electrophysiology, electroencephalography (EEG), and behavioral assessments were used to evaluate neurogenesis, microenvironment, circuit readouts, seizure burden, and cognition. NGF-hydrogel treatment was associated with preserved dentate gyrus neural stem cell populations, improved newborn granule cell localization and maturation, attenuated neuroinflammation and gliosis, and partial recovery of inhibitory interneuron markers. These changes were accompanied by improved hippocampal circuit readouts, reduced chronic spontaneous seizure burden, and enhanced recognition and spatial memory. Our findings indicate that local NGF-hydrogel delivery following status epilepticus is associated with improved hippocampal remodeling and functional outcomes, and suggest that biomaterial-based neurotrophic support may be a promising strategy for providing targeted neuroprotection and facilitating excitatory/inhibitory (E/I) balance reconstruction in the epileptic hippocampus.

## 1. Introduction

TLE is one of the most common forms of focal epilepsy in adults, and mesial temporal lobe epilepsy is frequently associated with hippocampal sclerosis. In addition to recurrent spontaneous seizures, patients with TLE often develop cognitive and neuropsychiatric comorbidities, including memory impairment, executive dysfunction, anxiety, and depression, all of which substantially worsen quality of life and long-term prognosis [1,2,3,4]. Although the range of available anti-seizure medications has expanded, a substantial proportion of patients still fail to achieve sustained seizure control. Clinical follow-up studies have shown that patients who eventually achieve good seizure control usually respond within the first two anti-seizure medication regimens, whereas further treatment adjustments provide only limited additional benefit [5]. These observations indicate that current therapies remain largely focused on symptomatic seizure suppression and have limited capacity to modify the pathological processes that drive epileptogenesis and disease progression.

TLE is widely considered to arise from, and be sustained by, progressive structural remodeling and functional dysregulation within hippocampal networks, with disruption of excitation-inhibition balance representing a central pathogenic feature [6]. Under physiological conditions, the dentate gyrus serves as a critical gate that limits the transfer of excitatory input from the entorhinal cortex to downstream hippocampal circuits. Once this gating function is compromised, aberrant excitation is more likely to spread through the hippocampal network and contribute to epileptic activity [6]. In parallel, loss or dysfunction of inhibitory interneurons, granule cell layer reorganization, aberrant mossy fiber sprouting, and altered local connectivity collectively promote the establishment of a hyperexcitable network state [6,7,8]. Among these inhibitory components, parvalbumin-positive interneurons are essential for maintaining temporal precision and network synchrony, whereas neuropeptide Y-related signaling has been implicated in endogenous anti-epileptiform regulation [7,9].

The dentate gyrus is one of the principal neurogenic niches in the adult hippocampus. After seizures, adult hippocampal neurogenesis becomes profoundly dysregulated, manifesting not only as altered proliferative activity, but also as ectopic migration of newborn granule cells, abnormal dendritic development, and inappropriate synaptic incorporation. These abnormalities contribute to maladaptive circuit remodeling and are closely linked to epileptogenesis and disease progression [10,11,12]. In parallel, glial activation and persistent inflammatory responses further disrupt the neurogenic microenvironment. Microglia, astrocytes, and complement-associated signaling pathways participate in synaptic remodeling and the establishment of aberrant network activity. Notably, sustained activation of the hippocampal C1q-C3 signaling cascade after status epilepticus has been associated with subsequent seizure burden [13,14]. Together, these observations suggest that seizure suppression alone is unlikely to adequately address the progressive pathological remodeling that follows epilepsy. Instead, strategies aimed at improving the quality of neurogenesis, limiting inflammation-associated injury, and preserving inhibitory circuit integrity may offer a more relevant framework for providing robust neuroprotection and facilitating E/I balance reconstruction.

Nerve growth factor (NGF), a key member of the neurotrophin family, plays an important role in neuronal survival, synaptic plasticity, and cognitive function [15,16]. Previous studies have shown that increasing NGF levels in the hippocampus can enhance neural plasticity and improve related behavioral outcomes. In addition, NGF contributes to the maintenance of normal hippocampal GABAergic circuit function [17,18,19]. These properties suggest that NGF may be relevant to the regulation of neurogenesis-associated remodeling and circuit stability in epilepsy. However, the therapeutic application of NGF remains substantially limited by its short half-life and poor penetration across the blood–brain barrier. Systemic administration is also associated with peripheral adverse effects, including hyperalgesia and weight loss, whereas local administration alone generally provides only transient exposure [19,20]. Hydrogels, by enabling sustained and relatively stable local release of bioactive molecules, offer a feasible strategy for targeted intracerebral delivery. In this context, NGF-hydrogel may provide a practical approach for improving local neurotrophic support within the epileptic hippocampus [21].

Hydrogels have emerged as highly promising delivery systems for treatments targeting the central nervous system (CNS). Due to their high water content and three-dimensional porous structure, hydrogels closely mimic the natural extracellular matrix, offering excellent biocompatibility and enabling the sustained, localized release of therapeutic biological agents. Furthermore, when combined with stereotaxic injection, injectable hydrogel systems can effectively bypass the blood–brain barrier (BBB) to achieve precise drug delivery directly to the lesion site [22]. However, their clinical application is frequently hindered by several inherent challenges, including potential mechanical mismatch with soft brain tissues, poor control over release rates, and the risk of initial burst release of the encapsulated agents [23], which can lead to suboptimal therapeutic efficacy and local toxicity [24]. To address these limitations, the present study utilizes an optimized injectable hydrogel system with finely tuned mechanical properties that match the hippocampal microenvironment. This design minimizes mechanical stress during administration and ensures the stable, long-term local delivery of NGF to the epileptogenic zone, thereby maximizing its neuroprotective effects and facilitating the reconstruction of the excitatory/inhibitory (E/I) balance in the dentate gyrus.

On this basis, we locally administered NGF-hydrogel into the hippocampus during the latent phase of epileptogenesis in a kainic acid-induced mouse model of epilepsy. The primary objective of this study was to investigate the efficacy of the NGF-based hydrogel in promoting robust neuroprotection and facilitating excitatory/inhibitory (E/I) balance reconstruction in the hippocampal dentate gyrus (DG). To this end, we systematically examined its effects on adult hippocampal neurogenesis, inhibitory interneuron-related populations, and the local inflammatory microenvironment. Furthermore, by specifically evaluating the synaptic integration of Syn1 and PSD95 within BrdU^+^/Map2^+^ newborn neurons, along with long-range tracing, planar microelectrode array recordings, and in vivo video-EEG monitoring, we aimed to determine whether this targeted intervention could effectively attenuate epileptic seizure severity. Ultimately, this study seeks to provide a promising translational strategy for the clinical management of refractory epilepsy.

## 2. Materials and Methods

### 2.1. Animal

All animal procedures were approved by the Laboratory Animal Center of Capital Medical University and were conducted in strict accordance with the regulations of the center and the Beijing Association for Laboratory Animal Science (Ethics Approval Number: AEEI-2025-867). Two types of male mice were used in this study: wild-type C57BL/6J mice aged 8–12 weeks, and Nestin-CreERT2;Rosa26-tdTomato transgenic mice on a C57BL/6J background, which were used for specific labeling of neural stem cells and their progeny. All animals were housed in a specific pathogen-free facility under controlled environmental conditions, with a temperature of 21–23 °C, relative humidity of 50–60% and a 12-h light/12-h dark cycle. Food and water were provided ad libitum. All mice were allowed to acclimatize for at least 1 week before the start of the experiments.

### 2.2. Preparation and Properties of the NGF-Hydrogel

The NGF-hydrogel was prepared using a thermosensitive chitosan-based formulation. Briefly, chitosan was dissolved in an acidic solution, followed by the precise addition of β-glycerophosphate (β-GP) at 4 °C to form the polymer precursor. Crucially, the incorporation of β-GP allowed the pH of the precursor solution to be rigorously adjusted and maintained within the physiological range (pH ~7.4) without polymer precipitation, ensuring optimal biocompatibility and minimizing local tissue irritation in the central nervous system. Subsequently, recombinant mouse NGF (100 ng/mL) was uniformly dispersed into the precursor solution under continuous gentle stirring at 4 °C.

Regarding its physical characteristics and premature stability, the formulated hydrogel precursor demonstrates excellent stability, maintaining a stable liquid (sol) state when stored at 4 °C. This property ensures smooth extrudability through a micro-syringe during stereotactic surgery without premature clogging. Upon reaching physiological temperature (37 °C) immediately following intracerebral injection, the formulation rapidly undergoes a temperature-dependent phase transition to form a stable three-dimensional hydrogel network. This rapid in situ gelation prevents the premature diffusion and initial burst release of the encapsulated NGF, thereby establishing a stable local reservoir for sustained neurotrophic delivery to the epileptogenic zone. The concentration of recombinant mouse NGF (100 ng/mL) was carefully selected based on previous targeted delivery and classical neuroprotection studies [25,26], which demonstrated that this optimal dose provides robust neuroprotection and promotes local network repair in the CNS without inducing overt cellular toxicity or aberrant overgrowth.

### 2.3. Experimental Groups and Intervention Protocol

Initially, a total of 220 C57BL/6 mice aged 8–12 weeks were subjected to intrahippocampal kainic acid (KA) injection to induce the epilepsy model. During the acute status epilepticus (SE) phase, 28 mice died due to severe continuous seizures. Additionally, 18 mice were excluded from the study for failing to reach at least Racine stage IV seizures. The remaining 174 successfully established epileptic mice, along with an independent cohort of 52 sham-operated mice, constituted the final study cohort. A total of 226 successfully modeled or sham-operated mice were included and randomly assigned to three groups: (1) a saline control group, which received an equal volume of saline injected into the hippocampal CA1 region (*n* = 52); (2) an epilepsy model group, in which KA was injected into the hippocampal CA1 region to induce epilepsy (*n* = 88); and (3) an NGF-hydrogel treatment group, which received NGF-hydrogel administered into the hippocampal CA1 region beginning 3 days after KA injection, followed by repeated administration every 15 days until the end of the experiment (*n* = 86).

### 2.4. BrdU Administration

To label proliferating cells and trace their subsequent fate, all mice received intraperitoneal injections of 5-bromo-2′-deoxyuridine (BrdU; Sigma-Aldrich, St. Louis, MO, USA) for 7 consecutive days starting on day 3 after model induction or the corresponding treatment. BrdU was dissolved in 0.9% saline and administered at a dose of 50 mg/kg twice daily at 12-h intervals.

### 2.5. Establishment of the Epilepsy Model and Stereotactic Injection

A mouse model of TLE was established by stereotactic intrahippocampal injection of kainic acid (KA) as previously described [27,28]. Mice were anesthetized with inhaled isoflurane (3–5% for induction and 1–3% for maintenance) and secured in a stereotactic frame (RWD Life Science, Shenzhen, China) [29,30]. After routine skin preparation and disinfection, a midline scalp incision was made to expose the skull and bregma. Under sterile conditions, a 1-µL microsyringe connected to a microinjection pump (WPI Inc., Sarasota, FL, USA) was used to slowly deliver 500 nL of KA solution (0.5 mg/mL in saline; total dose, 0.25 µg) into the right hippocampal CA1 region. Stereotactic coordinates were determined according to the mouse brain atlas as follows: anteroposterior (AP), −2.0 mm from bregma; mediolateral (ML), −1.3 mm from the midline; and dorsoventral (DV), −1.6 mm below the dura [31]. The injection rate was 100 nL/min. After injection, the needle was left in place for 5 min to facilitate diffusion and minimize reflux, and was then slowly withdrawn [27]. Mice in the control group received an equal volume of saline at the same coordinates. To minimize postoperative pain and suffering, all mice (including both the KA and saline groups) received a subcutaneous injection of the analgesic meloxicam (5 mg/kg) immediately after the surgery and once daily for 3 consecutive days.

In the NGF-hydrogel treatment group, NGF-hydrogel was administered beginning 3 days after KA injection through a pre-implanted cannula targeting the ipsilateral hippocampal CA1 region. The injection volume was 1.5 µL per administration, delivered at a rate of 50 nL/min, and treatment was repeated every 15 days until the experimental endpoint. Before administration, the hydrogel was maintained at 4 °C in a liquid state. After injection into brain tissue, it underwent a temperature-dependent sol–gel transition, thereby enabling sustained local release of NGF [21].

### 2.6. Viral Tracing and Circuit Labeling

To assess the projection pattern of the perforant pathway from the entorhinal cortex (EC) to the hippocampal DG, an anterograde viral tracing experiment was performed 3 days before perfusion. Mice were anesthetized and positioned in a stereotactic frame, and the right EC was targeted according to the mouse brain atlas (anteroposterior (AP), −3.4 mm from bregma; mediolateral (ML), +4.7 mm from the midline; dorsoventral (DV), −2.8 mm below the dura). Under sterile conditions, 150 nL of the anterograde transsynaptic tracer H8-HSV-HBEGFP (titer > 1 × 10^9^ PFU/mL; Brain Case, Shenzhen, China) was slowly injected into the EC using a microinjection pump at a rate of 50 nL/min. After injection, the needle was left in place for 10 min to minimize reflux along the needle tract and was then slowly withdrawn.

After viral injection, mice were maintained for an additional 3 days before perfusion to allow anterograde transsynaptic spread within the neural circuit and expression of the fluorescent reporter. The H129 strain of HSV has been widely used for anterograde transsynaptic circuit tracing and enables selective labeling of downstream pathways connected to the injection site. In the present study, viral tracing signals were analyzed in conjunction with newborn neuron labeling to characterize circuit integration within the EC-DG pathway [32,33].

### 2.7. In Vivo Electrophysiological Monitoring

To assess the characteristics of spontaneous recurrent seizures during the chronic phase and to evaluate the effects of NGF-hydrogel treatment, simultaneous video-EEG monitoring was performed in awake, freely moving mice. At the time of stereotactic injection of KA or saline, a bipolar Teflon-coated stainless-steel recording electrode was implanted into the right hippocampal CA3 region (anteroposterior (AP), −2.9 mm from bregma; mediolateral (ML), +3.2 mm from the midline; dorsoventral (DV), −3.2 mm below the dura). Stainless-steel screws placed over the cerebellum and at bregma served as the reference and ground electrodes. After adequate postoperative recovery, mice were connected to a multichannel physiological signal acquisition system for continuous recording. Crucially, all recordings were conducted inside a grounded Faraday cage to eliminate external electromagnetic interference. EEG signals were sampled at 1 kHz and band-pass filtered between 0.3 and 100 Hz. Continuous recordings were specifically analyzed at exactly 33 days after epilepsy modeling. All acquired EEG data were decoded and analyzed using the NeuroExplorer software (version 5.452). It is important to note that our analysis was exclusively focused on macroscopic events; all seizure events were manually identified in conjunction with video recordings and classified as either focal seizures or generalized seizures. Interictal spike activity was not systematically recorded or quantitatively analyzed in this current study. Focal seizures were defined as localized epileptiform discharges lasting longer than 10 s and corresponding to Racine stage 1–3 behavioral manifestations. Generalized seizures were defined as EEG discharges with amplitudes exceeding three times baseline for more than 10 s, accompanied by prominent postictal suppression, and corresponding to Racine stage 4–5 behavioral manifestations. All mice included in the analysis exhibited spontaneous recurrent seizures during the chronic phase. The frequency and duration of each seizure type were quantified separately to systematically evaluate the effects of NGF-hydrogel on chronic seizure burden.

### 2.8. Tamoxifen-Induced Lineage Tracing

For Nestin-CreERT2;Rosa26-tdTomato transgenic mice, a tamoxifen induction strategy was used for lineage labeling of neural stem cells and their progeny. Tamoxifen (Sigma-Aldrich) was dissolved in corn oil to prepare a 20 mg/mL stock solution [34], dissolved at 37 °C with shaking in the dark, and stored at 4 °C until use [35]. At 8 weeks of age, mice received intraperitoneal injections of tamoxifen once daily for 5 consecutive days before KA-induced model establishment, at a dose volume of 0.2 mL per 10 g body weight. Epilepsy model induction was performed 7 days after the final tamoxifen injection. This induction protocol effectively activates Nestin-CreERT2-mediated recombination, thereby enabling subsequent lineage tracing analysis of adult hippocampal neural stem cells [36].

### 2.9. Ex Vivo Planar Multielectrode Array Recording

To assess functional connectivity within local hippocampal networks, acute coronal hippocampal slices were prepared on day 30 after model induction, and field potential recordings were performed using a planar multielectrode array (MEA) system [37]. Mice were deeply anesthetized using an inhalation anesthesia system (induced with 3–4% isoflurane and maintained with 1.5–2% isoflurane delivered in a carrier gas of oxygen at a flow rate of 0.5–1.0 L/min via a precision vaporizer) and rapidly decapitated, and the brains were immediately removed and placed in ice-cold dissection solution continuously oxygenated with 95% O_2_/5% CO_2_. Coronal brain slices containing the hippocampus were cut at a thickness of 300 µm using a vibratome (Leica VT1000S, Wetzlar, Germany). Slices were then transferred to artificial cerebrospinal fluid (ACSF) maintained at 34 °C and continuously oxygenated for 60 min to allow recovery of tissue viability.

Electrophysiological recordings were performed using the MED64 planar multielectrode array system (MED-P515A, Alpha MED Sciences, Osaka, Japan), which consists of an 8 × 8 array of planar electrodes with an interelectrode spacing of 150 µm. During recording, slices were transferred to the recording chamber and continuously superfused with oxygenated ACSF at 32 °C at a flow rate of 2 mL/min. After placement on the array, slices were gently stabilized with a slice anchor. On the basis of hippocampal anatomy, electrodes covering the DG region were selected as the stimulation site and monophasic square pulses were delivered (pulse width, 0.2 ms). Field excitatory postsynaptic potentials (fEPSPs) were recorded simultaneously from multiple electrodes in the hippocampal CA1 region. Input–output (I/O) curves were generated by progressively increasing stimulus intensity to evaluate basal excitatory synaptic transmission and signal propagation within the local hippocampal network. Because coronal slices were used in this study, the recordings primarily reflected responses of local hippocampal circuits and field potential changes in the CA1 region.

To verify the synaptic nature of the recorded signals, ACSF containing 6-cyano-7-nitroquinoxaline-2,3-dione (CNQX) was applied at the end of recording to determine whether fEPSPs could be blocked. When necessary, ACSF containing tetrodotoxin (TTX) was further applied to assess the dependence of the recorded signals on action potential propagation. By comparing I/O curves and field potential response profiles across groups, we evaluated the effects of NGF-hydrogel on local hippocampal network function after epilepsy.

### 2.10. Behavioral Tests

All behavioral tests were conducted during the dark phase under quiet experimental conditions. To minimize stress, mice were handled by the experimenter for 5 min daily for 3 consecutive days before formal testing. On the test day, mice were transferred to the behavioral testing room 30 min in advance for habituation. After each trial, the apparatus was cleaned with 75% ethanol to eliminate potential olfactory cues.

The open field test (OFT) was used to assess anxiety-like behavior [38]. Mice were placed in the center of an opaque square arena (50 cm × 50 cm × 40 cm) and allowed to freely explore for 10 min. Locomotor activity was automatically recorded using a video-tracking system (Any-maze, Stoelting Co., Wood Dale, IL, USA). The arena floor was divided into a central zone (25 cm × 25 cm) and a peripheral zone. Time spent in the center and the number of center entries were used as the primary indices of anxiety-like behavior.

The novel object recognition (NOR) test was used to assess recognition memory [39]. The test consisted of three phases: habituation, training and testing. During habituation, performed 24 h before training, mice were allowed to freely explore the empty arena for 10 min. During training, two identical objects (A + A) were placed at opposite corners of the arena, and mice were allowed to explore freely for 10 min. During testing, conducted 24 h after training, one familiar object was replaced with a novel object (A + B), and the time spent exploring the familiar and novel objects was recorded over a 5-min session. Exploratory behavior was defined as the mouse directing its nose toward the object at a distance of less than 2 cm, or directly sniffing or touching the object. Climbing on, leaning against or walking around the object was not considered valid exploration. Recognition memory was evaluated using the discrimination index (*DI*DI) and the percentage of time spent exploring the novel object (%*Novel*), calculated as follows:

$$DI=\frac{\left(T_{\mathrm{novel}}-T_{\mathrm{familiar}}\right)}{\left(T_{\mathrm{novel}}+T_{\mathrm{familiar}}\right)};\%\mathrm{Novel}=\frac{T_{\mathrm{novel}}}{\left(T_{\mathrm{novel}}+T_{\mathrm{familiar}}\right)}\times 100\%$$

The Barnes maze was used to assess spatial learning and memory. The apparatus consisted of an elevated circular platform (90 cm in diameter, 1 m above the floor) with 20 evenly spaced holes (5 cm in diameter) around the perimeter. Only one hole was connected to an escape box and served as the target hole, whereas the remaining holes were empty. A bright overhead light (~400 lux) and white noise (85 dB) were used as aversive stimuli. Habituation was performed on day 0, during which mice were guided to the target hole and allowed to remain in the escape box for 2 min. Acquisition training was conducted on days 1–5, with four trials per day at 15-min intervals. At the start of each trial, mice were placed in a start chamber at the center of the platform. After 10 s, the chamber was removed and mice were allowed 180 s to locate the target hole. If a mouse failed to find the target hole within this period, it was gently guided to the escape box and allowed to remain there for 1 min. The probe trial was performed on day 6, with the escape box removed and all holes closed. Head-dip time at the target hole location during a 90-s session was recorded to assess spatial memory retention [40].

### 2.11. Statistical Analysis

Quantitative data are expressed as the mean ± standard error of the mean (SEM), except for seizure frequency data which are presented as the mean ± standard deviation (SD) to accurately reflect biological variance. Statistical analyses were performed using GraphPad Prism software (version 9). Prior to the application of any parametric tests, the data distribution was systematically evaluated for normality using the Shapiro–Wilk test, and the homogeneity of variances was confirmed using Levene’s test. The specific statistical testing models were meticulously selected to correspond directly with the experimental designs detailed in Section 3. For the comparison of macroscopic in vivo electrophysiological parameters (seizure counts) at 33 days post-modeling strictly between the epilepsy and NGF-hydrogel treatment groups, an unpaired two-tailed Student’s *t*-test was employed for normally distributed data, whereas the non-parametric Mann–Whitney U test was applied for non-normal count data. For single-factor comparisons among three experimental groups—which explicitly included the quantification of adult hippocampal neurogenesis markers, interneuron-related populations, local inflammatory cell densities, and the targeted evaluation of Syn1 and PSD95 synaptic markers specifically within BrdU+/Map2+ newborn neurons—a One-way Analysis of Variance (ANOVA) was employed. This was followed by Dunnett’s post hoc test when comparing treatment groups specifically to a single epilepsy control group, or Tukey’s post hoc test for comprehensive multiple pairwise comparisons. Conversely, for experimental data involving multiple longitudinal observation points—specifically the comprehensive behavioral assessments (Open Field Test, Novel Object Recognition, and Morris Water Maze) evaluated at 30 and 60 days—a Two-way ANOVA or repeated-measures Two-way ANOVA was utilized to assess the main effects and their interactions, followed by Bonferroni’s multiple comparisons test. A *p*-value of less than 0.05 was considered to indicate a statistically significant difference.

## 3. Results

### 3.1. Temporal Dynamics of Dentate Gyrus Remodeling in the KA-Induced TLE Model and Selection of an Early Intervention Time Point

**Figure 1:**
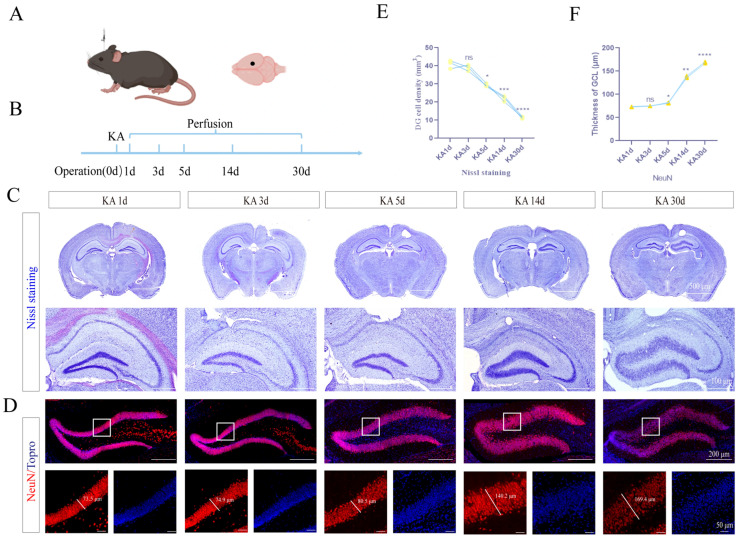
Temporal dynamics of dentate gyrus remodeling in the KA-induced TLE model and selection of an early intervention time point. (**A**) Schematic illustration of the experimental model and stereotactic KA injection into the unilateral hippocampus. (**B**) Experimental timeline. After KA injection, mice were perfused and brain tissues were collected at 1, 3, 5, 14, and 30 days for histological analysis. (**C**) Representative Nissl-stained images of the dentate gyrus at different time points after KA injection. Upper panels show coronal hippocampal sections; lower panels show higher-magnification views of the dentate gyrus. Scale bars: upper panels, 500 μm; lower panels, 100 μm. (**D**) Representative NeuN/Topro immunofluorescence images of the dentate gyrus at different time points after KA injection. Red fluorescence indicates NeuN-positive mature neurons, and blue fluorescence represents Topro nuclear counterstaining. The white boxed areas in the upper panels indicate the regions shown at higher magnification in the corresponding lower panels. Lower panels show higher-magnification views used for measurement of granule cell layer thickness. White lines indicate the measured thickness of the granule cell layer. Scale bars: upper panels, 200 μm; lower panels, 50 μm. (**E**) Statistical quantification of surviving neurons in the CA1 region across the indicated time points. Data are presented as the mean ± SEM. Statistical significance was determined by (One-way ANOVA *F*(4, 10) = 193.4, *p* < 0.0001). (**F**) Statistical quantification of NeuN-positive area/width across the indicated time points. Data are presented as the mean ± SEM. Statistical significance was determined by (One-way ANOVA *F*(4, 10) = 2495.0, *p* < 0.0001). Data are presented as the mean ± SEM. Here, *n* = 3 represents three independent biological replicates (individual mice) per group. For quantitative analysis, multiple brain slices and numerous target cells were meticulously captured and analyzed per mouse, and the mean value of each mouse was utilized as a single independent data point to strictly avoid pseudoreplication. *p* < 0.05 indicates a statistically significant difference. * *p* < 0.05, ** *p* < 0.01, *** *p* < 0.001, **** *p* < 0.0001; ns, not significant.

To establish a robust mouse model of TLE, kainic acid (KA) was stereotactically injected into the hippocampus (Figure 1A). This model induces status epilepticus and reproduces neuronal injury and structural remodeling resembling key pathological features of TLE [41,42,43]. To ensure model consistency, all animals were evaluated by simultaneous electroencephalographic (EEG) monitoring and Racine behavioral scoring, and only mice that developed stage IV or higher status epilepticus (SE) were included in subsequent analyses [44]. We then examined histopathological changes in the dentate gyrus (DG) at multiple time points after KA injection to characterize the temporal progression of hippocampal structural remodeling (Figure 1B).

Nissl staining revealed a time-dependent pattern of morphological changes in the DG after KA injection. Quantitative analysis of Nissl staining demonstrated a profound and progressive reduction in the density of surviving neurons within the hippocampal CA1 subfield across the longitudinal time points from day 1 to day 30 post-modeling (One-way ANOVA, *F*(4, 10) = 193.4, *p* < 0.0001). During the early stage after SE (days 1–3), the overall architecture of the DG remained largely preserved, and the granule cell layer retained a relatively compact and orderly laminar organization (Figure 1C), consistent with previous descriptions of early histopathological changes in KA models [28,45]. By day 5 after KA injection, structural abnormalities became evident, including widening of the granule cell layer and abnormal distribution of a subset of granule cells toward the molecular layer (Figure 1C). By day 30, the DG exhibited characteristic features of granule cell dispersion (GCD), with loosening of the granule cell layer, blurring of the boundary between the granule cell layer and molecular layer, and expansion of granule cell distribution (Figure 1C), in line with previous observations in experimental and human TLE [46,47].Quantitative evaluation of NeuN immunofluorescence demonstrated a highly significant, progressive increase across the evaluated longitudinal time points from day 1 to day 30 post-modeling (One-way ANOVA, *F*(4, 10) = 2495.0, *p* < 0.0001).

To further assess changes in neuronal organization, immunofluorescence staining for NeuN was performed at the corresponding time points (Figure 1D). Quantitative analysis showed a progressive decline in DG cell density based on Nissl staining (Figure 1E), accompanied by a progressive increase in granule cell layer thickness based on NeuN labeling (Figure 1F). These findings indicate that KA-induced hippocampal injury is associated with both reduced cellular density and expansion of the granule cell layer, consistent with previous reports of neuronal loss and structural reorganization in the epileptic dentate gyrus [10,48,49].

Taken together, these observations show that KA-induced DG pathology evolves in a time-dependent manner, with relatively preserved laminar structure during the early phase and progressive remodeling during subsequent stages. On the basis of this temporal profile, day 3 after KA injection was selected as an early intervention time point for subsequent NGF-hydrogel administration.

### 3.2. NGF-Hydrogel Is Associated with Preservation of Dentate Gyrus Stem Cell-Related Populations and Improved Positioning of Newborn Neurons After Epilepsy

**Figure 2:**
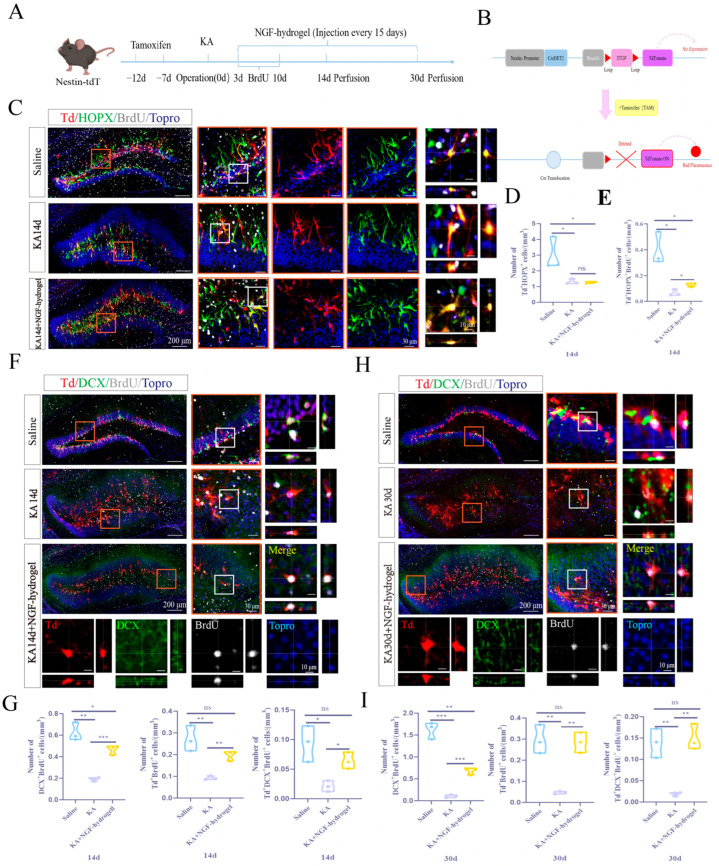
NGF-hydrogel is associated with preservation of dentate gyrus stem cell-related populations and improved positioning of newborn neurons after epilepsy. (**A**) Experimental timeline for *Nestin-CreERT2;Rosa26*-tdTomato transgenic mice. Tamoxifen (TAM) was administered before epilepsy induction to label Nestin-lineage cells. Epilepsy was induced by intrahippocampal KA injection at day 0. Beginning on day 3 after KA injection, BrdU was administered for 7 consecutive days, and NGF-hydrogel treatment was initiated and repeated every 15 days. Brain tissues were collected at days 14 and 30 after model induction. (**B**) Schematic illustration of the Nestin-lineage tracing strategy. Following TAM induction, Nestin-positive NSCs and their progeny were permanently labeled as tdTomato (Td)-positive cells. (**C**) Representative immunofluorescence images of HOPX/Td/BrdU/Topro staining in the DG of saline, KA14d, and KA14d + NGF-hydrogel groups. Left panels show low-magnification images of the DG. Middle panels show enlarged views of the boxed regions. Right panels show high-magnification confocal images with orthogonal views confirming colocalization. Scale bars: left panels, 200 μm; middle panels, 30 μm; right panels, 10 μm. (**D**,**E**) Quantification of Td^+^/HOPX^+^ cells (**D**) and Td^+^/HOPX^+^/BrdU^+^ cells (**E**) in the DG at day 14 after epilepsy induction. Statistical significance was determined by One-way ANOVA. For the proportion of Td^+^/HOPX^+^-positive cells (**D**), *F*(2, 6) = 7.44, *p* = 0.0237; for the proportion of Td^+^/HOPX^+^/BrdU^+^-positive proliferating cells (**E**), *F*(2, 6) = 16.29, *p* = 0.0038. (**F**) Representative immunofluorescence images of DCX/Td/BrdU/Topro staining in the DG of saline, KA14d, and KA14d + NGF-hydrogel groups. Left panels show low-magnification images of the DG. Middle panels show enlarged views of the boxed regions. Right panels show high-magnification confocal images with orthogonal views. Bottom panels show split-channel views of a representative Td^+^/DCX^+^/BrdU^+^ cell. Scale bars: left panels, 200 μm; middle panels, 30 μm; right panels and bottom panels, 10 μm. (**G**) Quantification of DCX^+^/BrdU^+^, Td^+^/BrdU^+^, and Td^+^/DCX^+^/BrdU^+^ cells in the DG at day 14 after epilepsy induction. (**H**) Representative immunofluorescence images of DCX/Td/BrdU/Topro staining in the DG of saline, KA30d, and KA30d + NGF-hydrogel groups. Left panels show low-magnification images of the DG. Middle panels show enlarged views of the boxed regions. Right panels show high-magnification confocal images with orthogonal views. Bottom panels show split-channel views of a representative Td^+^/DCX^+^/BrdU^+^ cell. Scale bars: left panels, 200 μm; middle panels, 30 μm; right panels and bottom panels, 10 μm. (**I**) Quantification of DCX^+^/BrdU^+^, Td^+^/BrdU^+^, and Td^+^/DCX^+^/BrdU^+^ cells in the DG at day 30 after epilepsy induction. Statistical significance was determined by One-way ANOVA. For parameters at day 14 (**G**): DCX/BrdU (*F*(2, 6) = 45.6, *p* = 0.0002), Td/BrdU (*F*(2, 6) = 17.6, *p* = 0.0031), and Td/DCX/BrdU (*F*(2, 6) = 10.0, *p* = 0.0123). For parameters at day 30 (**I**): DCX/BrdU (*F*(2, 6) = 119.2, *p* < 0.0001), Td/BrdU (*F*(2, 6) = 26.1, *p* = 0.0012), and Td/DCX/BrdU (*F*(2, 6) = 22.7, *p* = 0.0016). The orange boxed areas in the left panels indicate the regions shown at a higher magnification in the adjacent middle panels. The white boxed areas in the middle panels indicate the specific cells displayed at the highest magnification with orthogonal projections in the corresponding right panels. Data are presented as the mean ± SEM. Here, *n* = 3 represents three independent biological replicates (individual mice) per group. For quantitative analysis, multiple brain slices and numerous target cells were meticulously captured and analyzed per mouse, and the mean value of each mouse was utilized as a single independent data point to strictly avoid pseudoreplication. *p* < 0.05 indicates a statistically significant difference. * *p* < 0.05, ** *p* < 0.01, *** *p* < 0.001; ns, not significant.

To examine lineage-level changes in adult hippocampal neurogenesis after epilepsy, we performed fate mapping in Nestin-CreERT2;Rosa26-tdTomato transgenic mice. Following tamoxifen induction, Nestin-positive neural stem cells (NSCs) and their progeny were permanently labeled as tdTomato (Td)-positive cells, enabling longitudinal tracking of the neurogenic lineage [34,50] (Figure 2A,B). This strategy allowed us to assess neurogenesis-related alterations under epileptic conditions with reduced ambiguity in lineage identification.

To determine whether early NGF-hydrogel intervention affected stem cell-related populations after epilepsy, we first quantified HOPX and BrdU labeled cells within the Td^+^ lineage in the dentate gyrus (DG) at day 14 after status epilepticus (SE) (Figure 2C–E). HOPX was used as a marker associated with quiescent radial glia-like neural stem cells, whereas BrdU labeling was used to indicate proliferative activity. Compared with the saline group, the epilepsy group showed a significant reduction in both Td^+^/HOPX^+^ cells (One-way ANOVA, *F*(2, 6) = 7.44, *p* = 0.0237) and Td^+^/HOPX^+^/BrdU^+^ cells (One-way ANOVA, *F*(2, 6) = 16.29, *p* = 0.0038) in the DG, consistent with previous reports that epilepsy can drive abnormal activation and progressive depletion of the neural stem cell compartment [48,49,51,52]. Relative to the epilepsy group, NGF-hydrogel treatment significantly increased the numbers of Td^+^/HOPX^+^ cells and Td^+^/HOPX^+^/BrdU^+^ cells (*p* < 0.05), suggesting that NGF-hydrogel was associated with partial preservation of HOPX related stem cell-associated populations during the subacute stage after epilepsy.

We next examined whether NGF-hydrogel influenced the spatial distribution of downstream newborn neurons. To this end, the immature neuronal marker doublecortin (DCX) was analyzed together with Td and BrdU at days 14 and 30 after SE (Figure 2F–I). At day 14, the epilepsy group showed marked reductions in DCX^+^/BrdU^+^, Td^+^/BrdU^+^, and Td^+^/DCX^+^/BrdU^+^ cells compared with the saline group (Figure 2F,G). In addition, Td^+^/DCX^+^ cells in the epilepsy group displayed abnormal spatial distribution and disorganized orientation, with a subset of cells ectopically localized outside the subgranular zone (SGZ) and inner granule cell layer (GCL). These observations are consistent with previous studies showing that seizure-associated newborn granule cells can exhibit aberrant migration and abnormal structural development [2,46,53]. Compared with the epilepsy group, the NGF-hydrogel group showed significant increases in DCX^+^/BrdU^+^, Td^+^/BrdU^+^, and Td^+^/DCX^+^/BrdU^+^ cells (One-way ANOVA, *F*(2, 6) = 10.0, *p* = 0.0123) at day 14 (*p* < 0.05), and Td^+^/DCX^+^ cells were more frequently localized to the SGZ and inner GCL, with a more orderly orientation toward the molecular layer (Figure 2F,G).

Similar changes were observed at day 30 (Figure 2H,I). The epilepsy group remained markedly reduced in DCX^+^/BrdU^+^, Td^+^/BrdU^+^, and Td^+^/DCX^+^/BrdU^+^ cells (One-way ANOVA, *F*(2, 6) = 22.7, *p* = 0.0016) compared with the saline group, whereas NGF-hydrogel treatment significantly increased these indices relative to the epilepsy group (*p* < 0.05). Morphologically, Td^+^/DCX^+^ cells in the NGF-hydrogel group retained a distribution pattern more closely restricted to the SGZ/inner GCL, in contrast to the more ectopic and disorganized pattern observed in the untreated epilepsy group.

Taken together, these lineage-tracing and immunofluorescence data indicate that epilepsy was associated with reduced HOPX related stem cell-associated populations and abnormal positioning of DCX^+^ newborn neurons in the DG, whereas NGF-hydrogel treatment was associated with partial preservation of stem cell-related populations and improved spatial organization of newborn neurons.

### 3.3. NGF-Hydrogel Promotes Maturation-Related Phenotypes and Long-Term Retention of Newborn Neurons After Epilepsy

**Figure 3:**
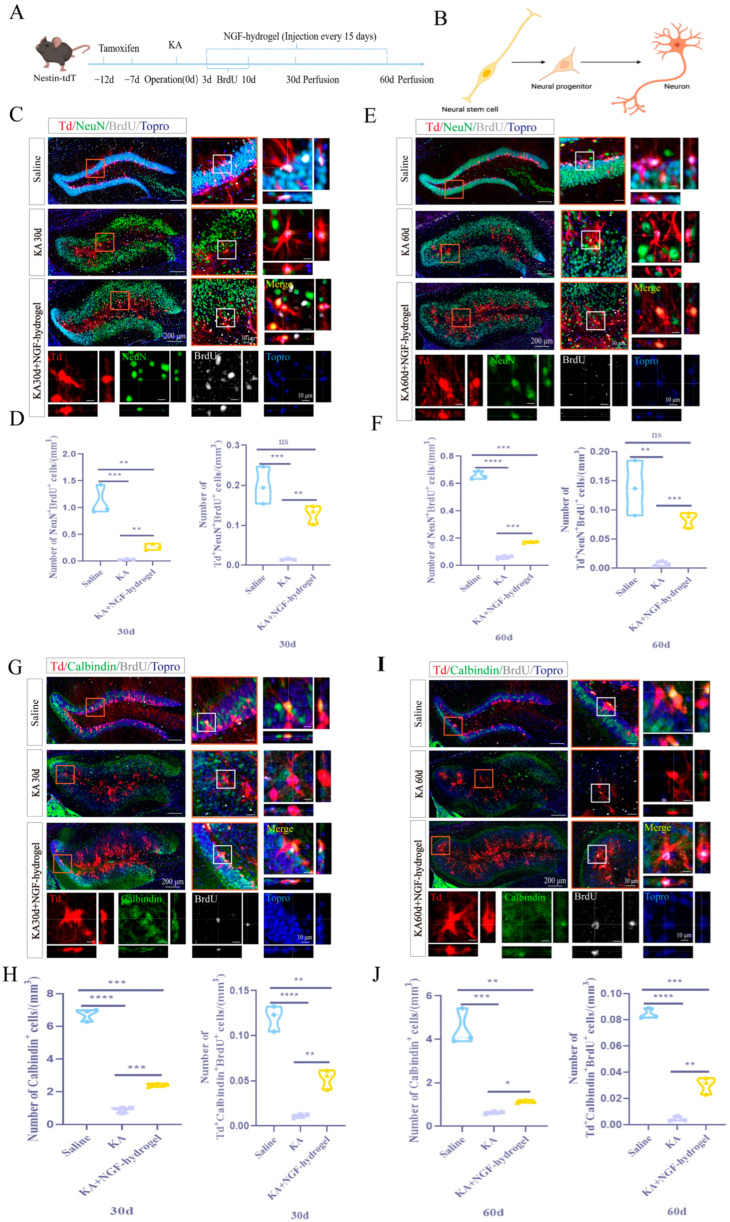
NGF-hydrogel promotes maturation-related phenotypes and long-term retention of newborn neurons after epilepsy. (**A**) Experimental timeline for Nestin-CreERT2;Rosa26-tdTomato mice. Tamoxifen was administered before epilepsy induction. Beginning on day 3 after KA injection, BrdU was administered for 7 consecutive days, and NGF-hydrogel treatment was initiated and repeated every 15 days. Brain tissues were collected at days 30 and 60 after model induction. (**B**) Schematic illustration of lineage progression from Nestin^+^ neural stem cells to mature neurons. (**C**) Representative immunofluorescence images of Td/NeuN/BrdU/Topro staining in the DG of saline, KA30d, and KA30d + NGF-hydrogel groups. Left panels show low-magnification images, middle panels show enlarged views of boxed regions, and right panels show high-magnification confocal images with orthogonal views. Bottom panels show split-channel views of a representative Td^+^/NeuN^+^/BrdU^+^ cell. Scale bars: left panels, 200 μm; middle panels, 30 μm; right panels and bottom panels, 10 μm. (**D**) Quantification of NeuN^+^/BrdU^+^ cells and Td^+^/NeuN^+^/BrdU^+^ cells in the DG at day 30 after epilepsy induction. (**E**) Representative immunofluorescence images of Td/NeuN/BrdU/Topro staining in the DG of saline, KA60d, and KA60d + NGF-hydrogel groups. Scale bars are the same as in (**C**). (**F**) Quantification of NeuN^+^/BrdU^+^ cells and Td^+^/NeuN^+^/BrdU^+^ cells in the DG at day 60 after epilepsy induction. Statistical significance was determined by One-way ANOVA. For parameters at day 30 (**D**): NeuN/BrdU (*F*(2, 6) = 36.15, *p* = 0.0004) and Td/NeuN/BrdU (*F*(2, 6) = 28.95, *p* = 0.0008). For parameters at day 60 (**F**): NeuN/BrdU (*F*(2, 6) = 794.6, *p* < 0.0001) and Td/NeuN/BrdU (*F*(2, 6) = 16.0, *p* = 0.0039). (**G**) Representative immunofluorescence images of Td/Calbindin/BrdU/Topro staining in the DG of saline, KA30d, and KA30d + NGF-hydrogel groups. Left panels show low-magnification images, middle panels show enlarged views of boxed regions, and right panels show high-magnification confocal images with orthogonal views. Bottom panels show split-channel views of a representative Td^+^/Calbindin^+^/BrdU^+^ cell. Scale bars: left panels, 200 μm; middle panels, 30 μm; right panels and bottom panels, 10 μm. (**H**) Quantification of total Calbindin^+^ cells and Td^+^/Calbindin^+^/BrdU^+^ cells in the DG at day 30 after epilepsy induction. (**I**) Representative immunofluorescence images of Td/Calbindin/BrdU/Topro staining in the DG of saline, KA60d, and KA60d + NGF-hydrogel groups. Scale bars are the same as in (**G**). (**J**) Quantification of total Calbindin^+^ cells and Td^+^/Calbindin^+^/BrdU^+^ cells in the DG at day 60 after epilepsy induction. Statistical significance was determined by One-way ANOVA. For parameters at day 30 (**H**): Calbindin (*F*(2, 6) = 408.1, *p* < 0.0001) and Td/Calbindin/BrdU (*F*(2, 6) = 81.8, *p* < 0.0001). For parameters at day 60 (**J**): Calbindin (*F*(2, 6) = 55.5, *p* = 0.0001) and Td/Calbindin/BrdU (*F*(2, 6) = 243.9, *p* < 0.0001). The orange boxed areas in the left panels indicate the regions shown at a higher magnification in the adjacent middle panels. The white boxed areas in the middle panels indicate the specific cells displayed at the highest magnification with orthogonal projections in the corresponding right panels. Data are presented as the mean ± SEM. Here, *n* = 3 represents three independent biological replicates (individual mice) per group. For quantitative analysis, multiple brain slices and numerous target cells were meticulously captured and analyzed per mouse, and the mean value of each mouse was utilized as a single independent data point to strictly avoid pseudoreplication. *p* < 0.05 indicates a statistically significant difference. * *p* < 0.05, ** *p* < 0.01, *** *p* < 0.001, **** *p* < 0.0001; ns, not significant.

To determine whether newborn cells generated after epilepsy could be retained and acquire a mature neuronal phenotype over time, we combined Nestin-lineage tracing with BrdU pulse labeling and NeuN immunostaining, and analyzed the DG at days 30 and 60 after status epilepticus (SE) (Figure 3A–F). This approach allowed identification of newborn neurons derived from the Nestin^+^ lineage that had undergone proliferation and subsequently expressed the mature neuronal marker NeuN (Td^+^/BrdU^+^/NeuN^+^) [48,54,55,56].

Compared with the saline group, the epilepsy group showed marked reductions in both NeuN^+^/BrdU^+^ cells and Td^+^/NeuN^+^/BrdU^+^ cells at days 30 and 60, indicating impaired retention and maturation of newborn neurons after epilepsy (Figure 3C–F). In contrast, NGF-hydrogel treatment significantly increased both indices relative to the epilepsy group at both time points (*p* < 0.05), suggesting that NGF-hydrogel was associated with improved long-term retention of newborn neurons and increased acquisition of a mature neuronal phenotype. These findings are consistent with previous studies showing that epilepsy can disrupt the maturation and integration of adult-born granule cells [2,48,57,58]. Long-term evaluation in Nestin-CreERT2/Rosa26-tdTomato transgenic mice revealed that NGF-hydrogel treatment significantly promoted the survival and functional maturation of newborn neurons. Specifically, the proportion of lineage-traced mature neurons (Td/NeuN/BrdU-positive) exhibited significant inter-group differences at both 30 days (One-way ANOVA, *F*(2, 6) = 28.95, *p* = 0.0008) and 60 days post-modeling (One-way ANOVA, *F*(2, 6) = 16.0, *p* = 0.0039).

We next examined calbindin-D28k (Calbindin), a marker associated with mature dentate granule cells, in combination with Td and BrdU at days 30 and 60 after SE (Figure 3G–J). The epilepsy group showed marked reductions in total Calbindin^+^ cells and Td^+^/Calbindin^+^/BrdU^+^ cells compared with the saline group. By contrast, NGF-hydrogel treatment significantly increased both indices at both time points relative to the epilepsy group (*p* < 0.05). Together, these results indicate that NGF-hydrogel treatment was associated with improved maturation-related phenotypes and sustained retention of newborn neurons in the epileptic DG. Further phenotypic analysis revealed that NGF-hydrogel administration significantly preserved Calbindin expression and promoted the specific differentiation of lineage-traced newborn cells into Calbindin-positive mature granule cells. Significant inter-group differences in the proportion of Td/Calbindin/BrdU-positive cells were observed at both 30 days (One-way ANOVA, *F*(2, 6) = 81.8, *p* < 0.0001) and 60 days post-modeling (One-way ANOVA, *F*(2, 6) = 243.9, *p* < 0.0001).

### 3.4. NGF-Hydrogel Is Associated with Preservation of NPY^+^ and PV^+^ Interneuron-Related Markers After Epilepsy

**Figure 4:**
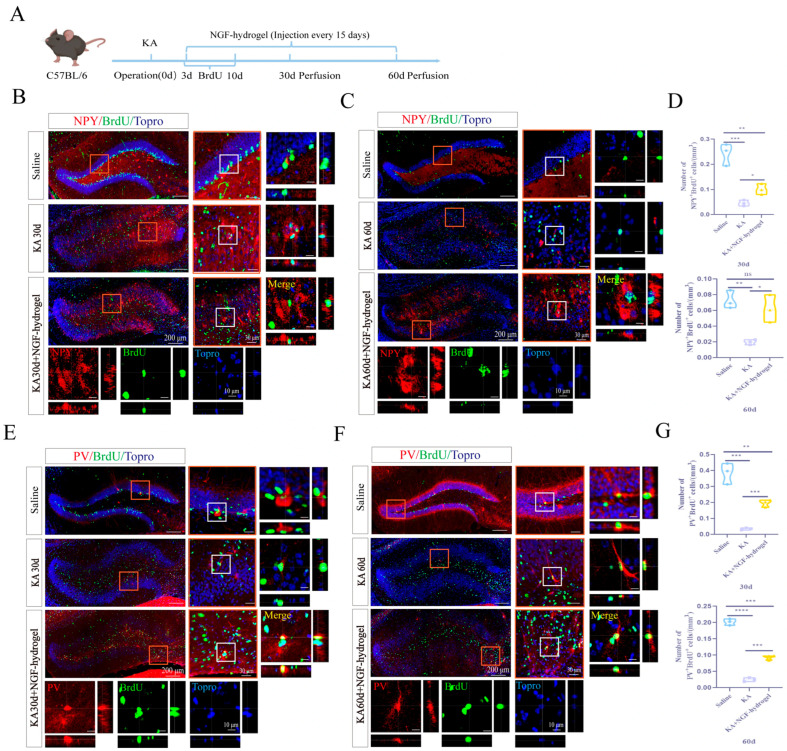
NGF-hydrogel is associated with preservation of NPY+ and PV+ interneuron-related markers after epilepsy. (**A**) Experimental timeline for C57BL/6J mice. Beginning on day 3 after KA injection, BrdU was administered for 7 consecutive days, and NGF-hydrogel treatment was initiated and repeated every 15 days. Brain tissues were collected at days 30 and 60 after model induction. (**B**,**C**) Representative immunofluorescence images of NPY/BrdU/Topro staining in the hippocampus of saline, KA30d, KA30d + NGF-hydrogel (**B**), saline, KA60d, and KA60d + NGF-hydrogel groups (**C**). Left panels show low-magnification images, middle panels show enlarged views of boxed regions, and right panels show high-magnification confocal images with orthogonal views. Bottom panels show split-channel views of a representative NPY^+^/BrdU^+^ cell. Scale bars: left panels, 200 μm; middle panels, 30 μm; right panels and bottom panels, 10 μm. (**D**) Quantification of NPY^+^/BrdU^+^ cells in the hippocampus at days 30 and 60 after epilepsy induction. For NPY/BrdU parameters (**D**) day 30 (*F*(2, 6) = 36.6, *p* = 0.0004) and day 60 (*F*(2, 6) = 15.8, *p* = 0.0040).(**E**,**F**) Representative immunofluorescence images of PV/BrdU/Topro staining in the hippocampus of saline, KA30d, KA30d + NGF-hydrogel (**E**), saline, KA60d, and KA60d + NGF-hydrogel groups (**F**). Left panels show low-magnification images, middle panels show enlarged views of boxed regions, and right panels show high-magnification confocal images with orthogonal views. Bottom panels show split-channel views of a representative PV^+^/BrdU^+^ cell. Scale bars: left panels, 200 μm; middle panels, 30 μm; right panels and bottom panels, 10 μm. (**G**) Quantification of PV^+^/BrdU^+^ cells in the hippocampus at days 30 and 60 after epilepsy induction. For PV/BrdU parameters (**G**): day 30 (*F*(2, 6) = 55.5, *p* = 0.0001) and day 60 (*F*(2, 6) = 381.7, *p* < 0.0001). Red fluorescence indicates NPY- or PV-positive interneurons, green fluorescence represents BrdU-positive newborn cells, and blue fluorescence indicates Topro nuclear counterstaining.The orange boxed areas in the left panels indicate the regions shown at a higher magnification in the adjacent middle panels. The white boxed areas in the middle panels indicate the specific cells displayed at the highest magnification with orthogonal projections in the corresponding right panels. Data are presented as the mean ± SEM. Here, *n* = 3 represents three independent biological replicates (individual mice) per group. For quantitative analysis, multiple brain slices and numerous target cells were meticulously captured and analyzed per mouse, and the mean value of each mouse was utilized as a single independent data point to strictly avoid pseudoreplication. *p* < 0.05 indicates a statistically significant difference. * *p* < 0.05, ** *p* < 0.01, *** *p* < 0.001, **** *p* < 0.0001; ns, not significant.

To assess whether NGF-hydrogel affected inhibitory interneuron-related changes after epilepsy, we examined NPY and PV in combination with BrdU at days 30 and 60 after SE (Figure 4A–G).

At day 30, the epilepsy group showed a marked reduction in NPY^+^/BrdU^+^ cells compared with the saline group, whereas NGF-hydrogel treatment significantly increased this index relative to the epilepsy group (Figure 4B,D). A similar pattern was observed at day 60 (Figure 4C,D). Quantitative analysis demonstrated that NGF-hydrogel treatment significantly preserved the population of newborn NPY/BrdU-positive inhibitory interneurons at both 30 days (One-way ANOVA, *F*(2, 6) = 36.6, *p* = 0.0004) and 60 days post-modeling (One-way ANOVA, *F*(2, 6) = 15.8, *p* = 0.0040) (Figure 4D). Morphologically, NPY-associated signals were markedly reduced in the untreated epilepsy group, whereas the NGF-hydrogel group showed a stronger retained signal pattern. These findings are consistent with previous reports that epilepsy is associated with impairment of NPY-related inhibitory components [9,59].

We next examined PV-related changes. The epilepsy group showed a marked reduction in PV^+^/BrdU^+^ cells at both day 30 and day 60 compared with the saline group (Figure 4E–G). In contrast, NGF-hydrogel treatment significantly increased PV^+^/BrdU^+^ cell numbers at both time points relative to the epilepsy group. The proportion of PV/BrdU-positive interneurons exhibited significant inter-group differences, with NGF-hydrogel exerting a robust protective effect on this specific neuronal subpopulation at day 30 (One-way ANOVA, *F*(2, 6) = 55.5, *p* = 0.0001) and day 60 (One-way ANOVA, *F*(2, 6) = 381.7, *p* < 0.0001) (Figure 4G).These findings are in line with previous studies showing selective vulnerability of PV-related inhibitory components in epilepsy [56,60,61].

Together, these data indicate that NGF-hydrogel treatment was associated with partial preservation of NPY and PV related inhibitory markers in the epileptic hippocampus.

### 3.5. NGF-Hydrogel Attenuates Microglial Activation, GFAP-Related Changes, and Neuronal Lamp1-Associated Burden After Epilepsy

**Figure 5:**
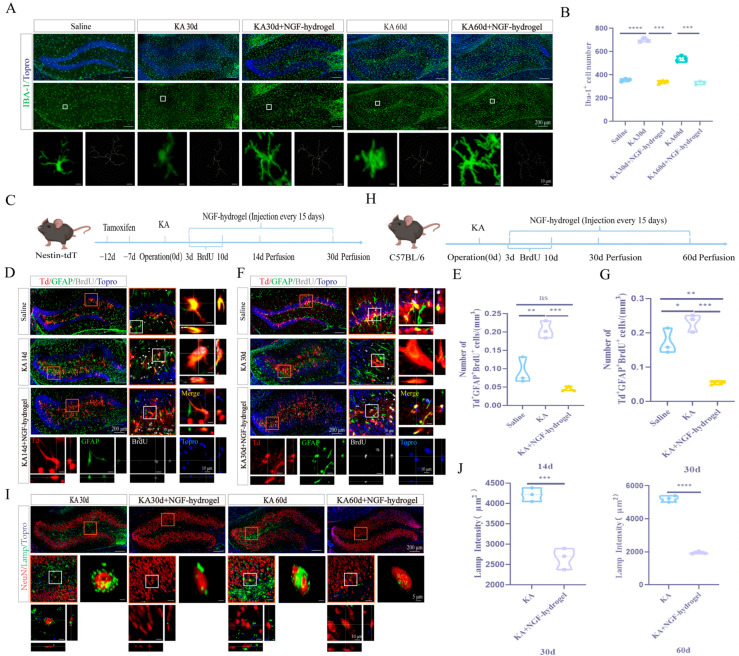
NGF-hydrogel attenuates microglial activation, GFAP-related changes, and neuronal Lamp1-associated burden after epilepsy. (**A**) Representative immunofluorescence images of IBA-1/Topro staining in the hippocampus of saline, KA30d, KA30d + NGF-hydrogel, KA60d, and KA60d + NGF-hydrogel groups. Upper panels show low-magnification images, middle panels show enlarged views of boxed regions, and lower panels show representative high-magnification images of microglial morphology together with Sholl analysis. Scale bars: upper and middle panels, 200 μm; lower panels, 10 μm. (**B**) Quantification of IBA-1^+^ cell numbers in the hippocampus at the indicated time points. For IBA-1 quantification (**B**): *F*(4, 10) = 255.2, *p* < 0.0001. (**C**) Experimental timeline for analysis of Td/GFAP/BrdU staining in Nestin-CreERT2;Rosa26-tdTomato mice. (**D**) Representative immunofluorescence images of Td/GFAP/BrdU/Topro staining in the DG of saline, KA14d, and KA14d + NGF-hydrogel groups. Left panels show low-magnification images, middle panels show enlarged views of boxed regions, right panels show high-magnification confocal images with orthogonal views, and bottom panels show split-channel views. Scale bars: left panels, 200 μm; middle panels, 30 μm; right and bottom panels, 10 μm. (**E**) Quantification of Td^+^/GFAP^+^/BrdU^+^ cells in the DG at day 14 after epilepsy induction. (**F**) Representative immunofluorescence images of Td/GFAP/BrdU/Topro staining in the DG of saline, KA30d, and KA30d + NGF-hydrogel groups. Scale bars are the same as in (**D**). (**G**) Quantification of Td^+^/GFAP^+^/BrdU^+^ cells in the DG at day 30 after epilepsy induction. (**H**) Experimental timeline for analysis of NeuN/Lamp1 staining in C57BL/6J mice. For Td/GFAP/BrdU parameters: day 14 (**E**) (*F*(2, 6) = 32.65, *p* = 0.0006) and day 30 (**G**) (*F*(2, 6) = 35.27, *p* = 0.0005). (**I**) Representative immunofluorescence images of NeuN/Lamp1/Topro staining in the DG of KA30d, KA30d + NGF-hydrogel, KA60d, and KA60d + NGF-hydrogel groups. Upper panels show low-magnification images, middle panels show enlarged views of boxed regions, and lower panels show high-magnification views of representative Lamp1^+^ puncta in NeuN^+^ neurons. Scale bars: upper panels, 200 μm; middle panels, 5 μm; lower panels, 10 μm. (**J**) Quantification of Lamp1 intensity at days 30 and 60 after epilepsy induction. Statistical significance for Lamp/NeuN (**J**) was determined by Unpaired Student’s *t*-test: day 30 (*t*(4) = 8.68, *p* = 0.0010) and day 60 (*t*(4) = 27.78, *p* < 0.0001) (*n* = 3). * *p* < 0.05, ** *p* < 0.01, *** *p* < 0.001; **** *p* < 0.0001; ns, not significant. Green fluorescence represents IBA-1, GFAP, or Lamp1; red fluorescence indicates Td, NeuN, or Tuj1; white fluorescence represents BrdU-positive cells; and blue fluorescence indicates Topro nuclear counterstaining. The orange boxed areas indicate regions shown at a higher magnification. The white boxed areas indicate specific cells displayed at the highest magnification, including orthogonal projections.

To assess the effects of NGF-hydrogel on the hippocampal microenvironment after epilepsy, we examined IBA-1, GFAP, and Lamp1 at different time points after SE (Figure 5A–J).

IBA-1 staining showed that the epilepsy group exhibited increased numbers of IBA-1^+^ cells at both day 30 and day 60 compared with the saline group, together with a shift toward a more activated morphology characterized by enlarged somata and shortened processes (Figure 5A,B). In contrast, NGF-hydrogel treatment significantly reduced IBA-1^+^ cell numbers at both time points and was associated with a more ramified microglial morphology. Quantitative analysis of IBA-1 immunofluorescence revealed a significant reduction in microglial activation following NGF-hydrogel treatment across the observed time points (One-way ANOVA, *F*(4, 10) = 255.2, *p* < 0.0001) (Figure 5B). These findings are consistent with previous reports of persistent microglial activation in epileptic hippocampus [62,63].

We next examined GFAP-related changes within the Td lineage at days 14 and 30 (Figure 5C–G). The epilepsy group showed increased numbers of Td^+^/GFAP^+^/BrdU^+^ cells compared with the saline group, whereas NGF-hydrogel treatment significantly reduced this index at both time points (Figure 5D–G). In Nestin-CreERT2/Rosa26-tdTomato transgenic mice, lineage tracing analysis demonstrated that NGF-hydrogel treatment significantly inhibited aberrant astrocytic differentiation of neural precursors at both 14 days (One-way ANOVA, *F*(2, 6) = 32.65, *p* = 0.0006) and 30 days post-modeling (One-way ANOVA, *F*(2, 6) = 35.27, *p* = 0.0005) (Figure 5E,G). Morphologically, GFAP-associated signals in the epilepsy group appeared denser and more disorganized, while the NGF-hydrogel group showed a relatively less compact pattern.

Finally, we assessed Lamp1 signals in NeuN^+^ neurons at days 30 and 60 (Figure 5H–J). Compared with the epilepsy group, NGF-hydrogel treatment reduced Lamp1 intensity at both time points, and punctate Lamp1-associated structures appeared smaller and less aggregated. Furthermore, quantitative assessment of Lamp/NeuN levels indicated a significant therapeutic effect in the NGF-hydrogel group compared to the KA group at both 30 days (Unpaired Student’s *t*-test, *t*(4) = 8.68, *p* = 0.0010) and 60 days (Unpaired Student’s *t*-test, *t*(4) = 27.78, *p* < 0.0001) (Figure 5J). Together, these results indicate that NGF-hydrogel treatment was associated with attenuation of inflammatory and glial pathological changes, as well as reduced neuronal Lamp1-associated burden after epilepsy.

### 3.6. NGF-Hydrogel Improves Synaptic and Circuit-Related Readouts After Epilepsy

**Figure 6:**
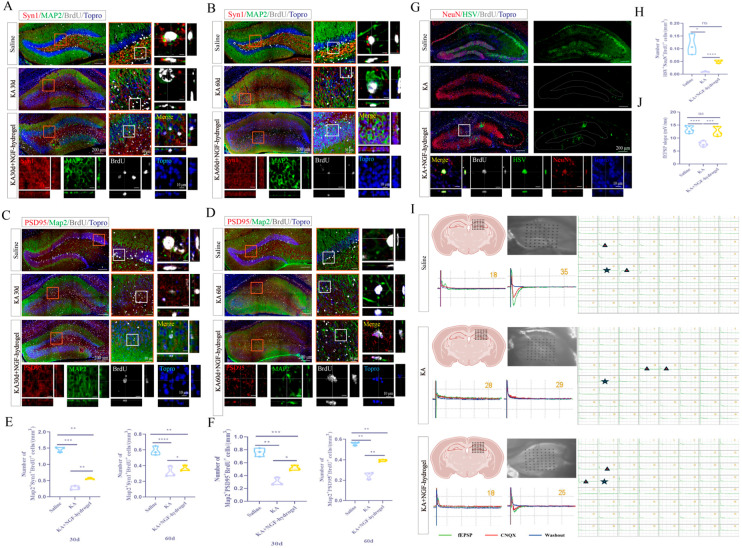
NGF-hydrogel improves synaptic and circuit-related readouts after epilepsy. (**A**) Representative immunofluorescence images of Syn1/Map2/BrdU/Topro staining in the DG of saline, KA30d, and KA30d + NGF-hydrogel groups. Left panels show low-magnification images, middle panels show enlarged views of boxed regions, and right panels show high-magnification images highlighting Syn1-associated puncta around BrdU^+^/Map2^+^ cells. Scale bars: left panels, 200 μm; middle panels, 30 μm; right panels, 10 μm. (**B**,**C**) Quantification of Syn1-related puncta around BrdU^+^/Map2^+^ cells at day 30 after epilepsy induction. (**D**) Representative immunofluorescence images of PSD95/Map2/BrdU/Topro staining in the DG of saline, KA60d, and KA60d + NGF-hydrogel groups. Left panels show low-magnification images, middle panels show enlarged views of boxed regions, and right panels show high-magnification images highlighting PSD95-associated puncta around BrdU^+^/Map2^+^ cells. Scale bars: left panels, 200 μm; middle panels, 30 μm; right panels, 10 μm. (**E**,**F**) Quantification of PSD95-related puncta around BrdU^+^/Map2^+^ cells at day 60 after epilepsy induction. For Map2/Syn1/BrdU parameters (**E**): day 30 (*F*(2, 6) = 14.6, *p* = 0.0050) and day 60 (*F*(2, 6) = 115.7, *p* < 0.0001). For Map2/PSD95/BrdU parameters (**F**): day 30 (*F*(2, 6) = 48.6, *p* = 0.0002) and day 60 (*F*(2, 6) = 19.0, *p* = 0.0025). (**G**) Schematic illustration of HSV-H129 injection into the EC for tracing of EC-DG pathway-related signals. (**H**) Representative images and quantification of GFP-positive tracing signals in the DG of saline, epilepsy, and NGF-hydrogel groups. For HSV circuit tracing quantification (**H**): *F*(2, 6) = 302.8, *p* < 0.0001. (**I**) Schematic illustration of MEA recording. The DG was stimulated and field responses were recorded in the hippocampal pathway. The star symbol (★) indicates the designated stimulation electrode site, and the triangles (▲) denote the corresponding recording electrode sites. (**J**) Quantification of fEPSP slope recorded by MEA in saline, epilepsy, and NGF-hydrogel groups. Statistical significance for fEPSP slopes (**J**) was determined by One-way ANOVA (*F*(2, 12) = 23.6, *p* < 0.0001, n = 5 per group). In the immunofluorescence images, red fluorescence indicates Syn1, PSD95, or NeuN; green fluorescence represents MAP2 or HSV; white fluorescence indicates BrdU-positive cells; and blue fluorescence represents Topro nuclear counterstaining. The orange boxed areas indicate regions shown at a higher magnification. The white boxed areas indicate specific cells displayed at the highest magnification with orthogonal projections. In the electrophysiological traces (Panel **I**), the green, red, and blue lines represent the baseline fEPSP, CNQX application, and washout phases, respectively. Data are presented as mean ± SEM. Statistical analysis was performed using one-way ANOVA followed by Dunnett’s T3 post hoc test (*n* = 3). * *p* < 0.05, ** *p* < 0.01, *** *p* < 0.001, **** *p* < 0.0001; ns, not significant.

To further determine whether the structural and cellular changes described above were accompanied by improvement in synaptic and circuit-related readouts, we analyzed synaptic marker distribution around BrdU-labeled newborn neurons and further assessed circuit-related changes by combining long-range tracing with ex vivo electrophysiological recordings [57,64,65] (Figure 6A–J).

We first examined Syn1 and PSD95 in relation to BrdU^+^/Map2^+^ cells in the DG. At day 30, immunofluorescence analysis showed that Syn1-associated punctate signals surrounding BrdU^+^/Map2^+^ cells were markedly reduced in the epilepsy group compared with the saline group (Figure 6A–C), suggesting impairment of presynaptic marker-associated structural readouts around newborn neurons under epileptic conditions. In contrast, NGF-hydrogel treatment significantly increased the density of Syn1-related puncta around BrdU^+^/Map2^+^ cells relative to the epilepsy group. Quantitative assessment of Map2/Syn1/BrdU co-labeling demonstrated that NGF-hydrogel treatment significantly promoted the formation of presynaptic terminals on newborn neurons at both 30 days (One-way ANOVA, *F*(2, 6) = 14.6, *p* = 0.0050) and 60 days post-modeling (One-way ANOVA, *F*(2, 6) = 115.7, *p* < 0.0001). We next assessed PSD95 using the same analytical strategy. At day 60, the epilepsy group exhibited a clear reduction in PSD95-associated puncta surrounding BrdU^+^/Map2^+^ cells, whereas NGF-hydrogel treatment significantly increased this index relative to the epilepsy group (Figure 6D–F). Similarly, the proportion of Map2/PSD95/BrdU-positive cells, indicative of postsynaptic specialization, exhibited significant inter-group differences, with the NGF-hydrogel group showing marked enhancement at both day 30 (One-way ANOVA, *F*(2, 6) = 48.6, *p* = 0.0002) and day 60 (One-way ANOVA, *F*(2, 6) = 19.0, *p* = 0.0025). Together, these findings indicate that NGF-hydrogel treatment significantly enhanced the synaptic integration of BrdU^+^/Map2^+^ newborn neurons, providing a crucial structural basis for E/I balance reconstruction and counteracting the seizure-induced disruption of adult-born granule cell maturation [57,64].

To further examine whether these local synaptic changes were accompanied by improvement in long-range circuit-related readouts, we performed EC injection of HSV-H129 and assessed GFP-positive tracing signals in the DG [33,66] (Figure 6G,H). In the epilepsy group, GFP-positive labeling in the DG was reduced and appeared less continuous than in the saline group, indicating impaired EC-DG pathway-related tracing signals after epilepsy. By contrast, the NGF-hydrogel group showed stronger and more broadly distributed GFP-positive signals in the DG than the epilepsy group, suggesting improvement in EC-DG pathway-related labeling [33,66,67] (One-way ANOVA, *F*(2, 6) = 302.8, *p* < 0.0001).

Finally, we assessed hippocampal pathway transmission using MEA. With stimulation delivered to the DG and field responses recorded in CA1, the epilepsy group showed a marked reduction in fEPSP slope compared with the saline group (Figure 6I,J), indicating impaired pathway transmission after epilepsy. Compared with the epilepsy group, NGF-hydrogel treatment significantly increased fEPSP slope (One-way ANOVA, *F*(2, 12) = 23.6, *p* < 0.0001), suggesting partial recovery of hippocampal pathway transmission. This interpretation is consistent with the use of fEPSP slope as an index of synaptic transmission efficiency in slice-based electrophysiological analysis [1].

Overall, these data show that NGF-hydrogel treatment was associated with improvement in synaptic marker-associated structural readouts around newborn neurons, enhanced EC-DG pathway-related tracing signals, and partial recovery of MEA-recorded pathway transmission after epilepsy.

### 3.7. NGF-Hydrogel Reduces Chronic Seizure Burden and Improves Affective and Cognitive Behavioral Outcomes After Epilepsy

**Figure 7:**
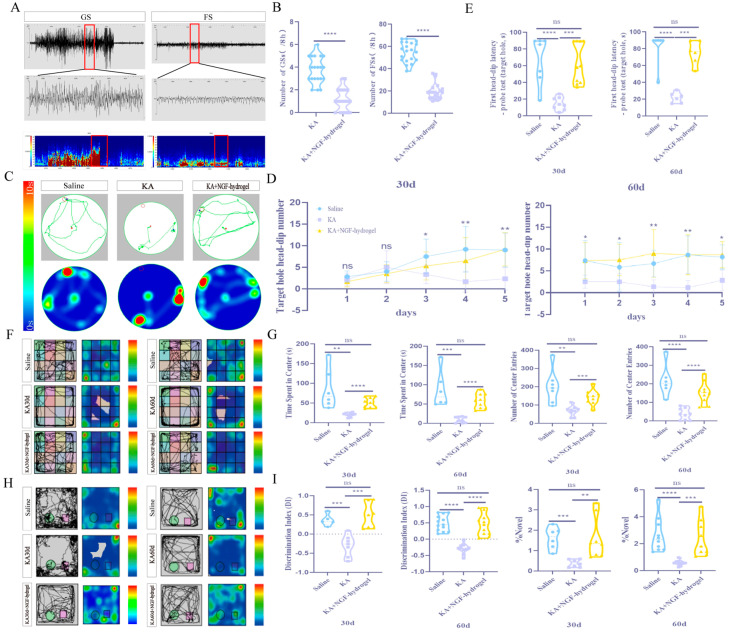
NGF-hydrogel reduces chronic seizure burden and improves affective and cognitive behavioral outcomes after epilepsy. (**A**) Representative in vivo EEG traces showing GS and FS recorded during the chronic stage after model induction. Red boxes indicate enlarged regions shown below each trace. The red boxed areas in Panel A indicate the specific time windows of the EEG traces and spectrograms that are shown at a higher temporal resolution (magnified view) in the panels immediately below. (**B**) Quantification of the frequency of GS and FS in the epilepsy and NGF-hydrogel groups at day 33 after model induction. *n* = 8 mice per group. Statistical significance for GS and FS (**B**) was determined by Unpaired Student’s *t*-test (*n* = 20 mice per group) (**C**) Representative trajectories and spatial distributions of mice from the saline, epilepsy, and NGF-hydrogel groups in the Barnes maze test. (**D**) Quantification of target hole head-dip number during Barnes maze training at days 30 and 60 after model induction. (**E**) Quantification of first head-dip latency at the target hole during the Barnes maze probe test at days 30 and 60 after model induction. For Barnes maze parameters (**E**): day 30 (*n* = 7, 8, 10 for Saline, KA, and KA + NGF groups, respectively) and day 60 (*n* = 6 per group). Statistical significance was determined by One-way ANOVA. (**F**) Representative trajectories and heatmaps of mice from each group in the OFT at days 30 and 60 after model induction. (**G**) Quantification of OFT performance, including time spent in the center and number of center entries, at days 30 and 60 after model induction. For OFT parameters (**G**), statistical significance was determined by One-way ANOVA. Exact *n* values varied slightly due to valid tracking: for center time (*n* = 6, 9, 9 at day 30; *n* = 6, 9, 9 at day 60) and for center entries (*n* = 7, 9, 9 at day 30; *n* = 6, 9, 10 at day 60). (**H**) Representative exploration trajectories and heatmaps from the NOR task in each group at days 30 and 60 after model induction. (**I**) Quantification of NOR performance, including DI and %Novel, at days 30 and 60 after model induction. For NOR discrimination index (**I**): day 30 (*n* = 5, 7, 7 for Saline, KA, and KA + NGF groups, respectively) and day 60 (*n* = 13, 10, 11, respectively). For the %Novel exploration parameter (**I**): day 30 (*n* = 6, 7, 8 for Saline, KA, and KA + NGF groups, respectively) and day 60 (*n* = 10, 10, 9, respectively). Statistical significance was determined by One-way ANOVA. Data are expressed as the mean ± standard deviation (SD) for seizure frequency/duration parameters, and as the mean ± standard error of the mean (SEM) for other behavioral/quantitative parameters. Statistical analysis was performed using one-way ANOVA followed by Dunnett’s T3 post hoc test. For in vivo EEG analysis, *n* = 8 mice per group. For behavioral analyses, *n* = 9 mice per group. * *p* < 0.05, ** *p* < 0.01, *** *p* < 0.001, **** *p* < 0.0001; ns, not significant.

To determine whether the histological, synaptic, and circuit-related changes described above were accompanied by improvement in disease-related functional outcomes, we performed in vivo video-EEG monitoring during the chronic stage and further assessed behavioral performance at days 30 and 60 after SE (Figure 7A–I).

At 33 days after model induction, representative EEG traces decoded by NeuroExplorer showed macroscopic generalized seizures (GS) and focal seizures (FS) in epileptic mice (Figure 7A); interictal spikes were not quantified. In vivo electrophysiological monitoring revealed that NGF-hydrogel treatment significantly attenuated seizure severity, evidenced by a marked reduction in both generalized seizures (GS) (Unpaired Student’s *t*-test, *t*(38) = 7.75, *p* < 0.0001) and focal seizures (FS) (Unpaired Student’s *t*-test, *t*(38) = 14.73, *p* < 0.0001) compared to the KA group. (Figure 7B). These findings indicate that NGF-hydrogel alleviated chronic spontaneous seizure burden after epilepsy, consistent with previous video-EEG studies showing that seizure frequency reflects the level of ongoing epileptogenic network activity [68,69].

We next performed the Barnes maze to assess spatial learning and memory. In the acquisition phase, the epilepsy group showed a lower number of target hole head-dips across training days at both day 30 and day 60, whereas the NGF-hydrogel group exhibited higher target hole head-dip numbers and a more favorable training profile (Figure 7C,D). In the probe trial, the epilepsy group displayed a shorter latency to the first head-dip at the target hole than the saline and NGF-hydrogel groups, while no significant difference was observed between the saline and NGF-hydrogel groups at either time point (Figure 7E). Spatial memory retention, assessed by the Barnes maze probe test, was significantly rescued by NGF-hydrogel administration at both 30 days (One-way ANOVA, *F*(2, 22) = 15.56, *p* < 0.0001) and 60 days post-modeling (One-way ANOVA, *F*(2, 15) = 29.56, *p* < 0.0001) [70,71,72].

To primarily assess general locomotor activity and baseline exploratory behavior, alongside affective states, we then performed the OFT at days 30 (One-way ANOVA, *F*(2, 21) = 11.16, *p* = 0.0005) and 60 (One-way ANOVA, *F*(2, 21) = 16.92, *p* < 0.0001). Representative trajectories and heatmaps showed that mice in the epilepsy group displayed reduced exploration of the center area, whereas NGF-hydrogel treatment increased center-directed exploration (Figure 7F). Quantitative analysis further showed that the epilepsy group spent less time in the center and made fewer center entries than the saline group, while NGF-hydrogel treatment significantly increased both indices relative to the epilepsy group at both time points (Figure 7G). These results indicate that NGF-hydrogel improved baseline exploratory behavior and partially ameliorated epilepsy-associated anxiety-like states, although the latter observation warrants cautious interpretation due to the reliance on a single behavioral paradigm [73,74,75].

We next used the NOR task to evaluate recognition memory. Representative exploration trajectories and heatmaps are shown in Figure 7H. Quantitative analysis demonstrated that the epilepsy group showed lower DI and lower %Novel than the saline group at both day 30 (One-way ANOVA, *F*(2, 16) = 15.53, *p* = 0.0002) (One-way ANOVA, *F*(2, 18) = 7.17, *p* = 0.0051) and day 60 (One-way ANOVA, *F*(2, 31) = 36.07, *p* < 0.0001) (One-way ANOVA, *F*(2, 26) = 11.13, *p* = 0.0003) (Figure 7I), indicating impaired recognition memory. In contrast, NGF-hydrogel treatment significantly increased both DI and %Novel relative to the epilepsy group at both time points, suggesting improved preference for the novel object and better recognition memory performance [76,77].

Overall, these results indicate that NGF-hydrogel treatment reduced chronic seizure burden and was associated with improvement in anxiety-like behavior, recognition memory, and Barnes maze-related performance after epilepsy.

## 4. Discussion

In the present study, local NGF-hydrogel administration during the latent phase after SE was associated with reduced chronic seizure burden and improved affective and cognitive behavioral performance in the KA-induced model. By integrating evidence from lineage tracing, immunofluorescence, synaptic marker analysis, long-range tracing, MEA recordings, in vivo EEG monitoring, and behavioral testing, our data suggest that the effects of NGF-hydrogel were not restricted to a single pathological process. Instead, NGF-hydrogel treatment was associated with coordinated improvement across several levels of epileptic hippocampal remodeling, including preservation of stem cell-related populations, improved maturation-related phenotypes of newborn neurons, partial preservation of inhibitory interneuron-related markers, attenuation of inflammatory and glial pathological changes, and improvement in synaptic and circuit-related readouts. Taken together, these findings support the view that early local neurotrophic intervention may improve the structural and functional state of the epileptic hippocampus beyond symptomatic seizure suppression alone.

A central finding of the present study is that NGF-hydrogel treatment was associated with improvement in several sequential aspects of adult hippocampal neurogenesis after epilepsy, including preservation of HOPX related stem cell-associated populations, improved positioning of DCX^+^ newborn neurons, and increased maturation-related phenotypes at later stages. In the epileptic hippocampus, pathological remodeling of adult neurogenesis is not simply reflected by a change in cell number; rather, it also involves depletion of the stem cell-associated compartment, ectopic migration of newborn granule cells, and disruption of their subsequent maturation trajectory. Previous studies have shown that HOPX marks a quiescent NSC-related population in the hippocampus and that seizure-related activity can drive abnormal activation and progressive depletion of this compartment [48,51]. In parallel, seizure-associated newborn neurons can undergo ectopic migration into the hilus and develop abnormal orientation and morphology, changes that are thought to contribute to maladaptive hippocampal remodeling [46,53]. Importantly, the pathological significance of aberrant neurogenesis in epilepsy lies not only in how many newborn cells are generated, but also in whether they are positioned appropriately and proceed along a more physiological maturation trajectory. In this regard, previous studies have emphasized that abnormal development and integration of adult-born granule cells can reshape hippocampal circuitry under epileptic conditions [2,57,58]. Our results are broadly consistent with this framework, as NGF-hydrogel treatment was associated with preservation of HOPX-related populations, reduced ectopic distribution of DCX^+^ cells, and increased NeuN- and Calbindin-related maturation indices at later time points, suggesting an overall shift toward a more organized neurogenic response after epilepsy.

Another notable finding of this study is that NGF-hydrogel treatment was associated not only with neurogenesis-related improvement, but also with partial preservation of inhibitory interneuron related markers and attenuation of inflammatory and glial pathological changes in the epileptic hippocampus. In epilepsy, disruption of the local inhibitory network and persistent inflammatory remodeling are closely intertwined processes. Previous studies have shown that PV related inhibitory components are particularly vulnerable under epileptic conditions, whereas NPY associated signaling is often regarded as an endogenous anticonvulsant mechanism within the hippocampus [9,56,59,60,61]. In parallel, activated microglia and reactive astrocytes contribute to synaptic remodeling, altered extracellular homeostasis, and sustained network hyperexcitability after seizures [62,63,78,79]. Our data are consistent with this general framework, as the untreated epilepsy group showed reduced NPY and PV related markers together with increased IBA-1 associated microglial activation, increased GFAP related changes, and enhanced Lamp1 associated neuronal burden. By contrast, NGF-hydrogel treatment was associated with partial preservation of inhibitory markers and a less reactive inflammatory-glial profile. Although the present study does not establish a direct causal relationship among these processes, it is reasonable to consider that preservation of a less inflammatory and less structurally disorganized microenvironment may be more permissive for orderly positioning and maturation of newborn neurons, while reduced microenvironmental stress and improved inhibitory support may together contribute to a more stable hippocampal network state.

The synaptic, circuit related, and functional findings in the present study further support the view that the effects of NGF-hydrogel extend beyond isolated cellular changes. In the epileptic hippocampus, abnormal development of newborn granule cells is thought to be particularly relevant because these cells can form inappropriate synaptic contacts and contribute to maladaptive circuit remodeling [57,58,64]. In this context, the increase in Syn1 and PSD95 related puncta surrounding BrdU^+^/Map2^+^ cells in the NGF-hydrogel group suggests that treatment was associated with improvement in synapse-related structural readouts around newborn neurons. At the circuit level, the stronger EC-DG pathway-related tracing signals and the increase in MEA recorded fEPSP slope further indicate that these local structural changes were accompanied by improvement in pathway-related readouts [1,33,66,67]. Notably, these synaptic and circuit related improvements were paralleled by reduced spontaneous seizure burden and better performance in OFT, NOR, and Barnes maze tasks. Although these data do not establish that improved newborn neuron maturation is solely responsible for the electrophysiological and behavioral benefits observed here, they do indicate a consistent relationship between local hippocampal remodeling and broader functional outcomes. From this perspective, NGF-hydrogel appears to promote a more favorable structural and functional state of the epileptic hippocampus, which may contribute to lower seizure burden and improved behavioral performance during the chronic stage.

Regarding the translational potential of this approach, the direct stereotaxic administration of the hydrogel into the hippocampus provides a highly viable clinical strategy for drug-resistant TLE. While invasive, targeting the epileptogenic zone directly is crucial because it completely bypasses the blood–brain barrier (BBB), which otherwise strictly limits the central availability of macromolecular biological agents like NGF. Furthermore, localized delivery effectively prevents the severe peripheral adverse effects typically associated with systemic NGF administration, such as hyperalgesia. In modern clinical neurosurgery [80], stereotaxic procedures are already routinely utilized for the management of refractory focal epilepsy. Techniques such as stereoelectroencephalography (SEEG) for precise seizure mapping, responsive neurostimulation (RNS), and localized interventions like laser interstitial thermal therapy (LITT) have become standard practices [81]. Therefore, utilizing an injectable hydrogel for sustained, in situ neurotrophic support precisely aligns with current minimally invasive neurosurgical trends, offering a highly practical avenue for translating targeted neuroprotection and E/I balance reconstruction into the clinical management of refractory epilepsy.

Several limitations of the present study should be acknowledged. First, although our data consistently showed that NGF-hydrogel treatment was associated with improvement in neurogenesis-related, synaptic, circuit-related, electrophysiological, and behavioral readouts, the present work does not establish a direct causal relationship among these levels of change. In particular, we did not perform newborn neuron-specific functional manipulation experiments to determine whether the observed improvements in seizure burden and behavioral performance were directly mediated by altered maturation or integration of adult-born granule cells. Second, the interpretation of PV^+^/BrdU^+^ and NPY^+^/BrdU^+^ cells should be made with caution, as these markers do not by themselves establish the generation of fully functional inhibitory interneurons. Additionally, while the OFT was utilized to gauge general locomotor activity and provided indices of anxiety-like states based on thigmotaxis, the absence of more specific affective tests such as the Elevated Plus Maze (EPM) represents a methodological limitation. Consequently, conclusions regarding anxiety-like behavior should be interpreted with caution. Finally, several analyses were performed with limited sample sizes, and only one experimental background was examined, which may restrict the generalizability of the findings. Accordingly, future studies incorporating more specific causal manipulation and broader validation will be important for further defining the therapeutic significance of NGF-hydrogel in epilepsy.

Importantly, the translational potential of such localized, biomaterial-based therapeutic strategies extends beyond the epileptic hippocampus. For instance, recent studies have demonstrated that the direct application of a nutritional gel containing neuronal growth factors and neural stem cells into the infarct cavity after the peak of stroke-induced neuroinflammation represents a highly feasible and effective approach to promote neurorestoration following cerebral ischemia [82]. Together with our findings, these approaches highlight a promising, overarching paradigm in which functionalized hydrogels can be leveraged to reconstruct damaged neural networks across various severe neurological disorders.

Crucially, these findings directly address the fundamental therapeutic deficiency identified in current clinical practice: the inability of conventional anti-seizure medications to halt progressive network deterioration. While standard therapies focus on acute symptomatic suppression, our data suggest that the sustained, localized delivery of NGF actively targets the underlying pathological substrate. By simultaneously conferring neuroprotection to vulnerable interneurons and facilitating the proper synaptic integration of newborn neurons, the NGF-hydrogel intervention acts on the critical structural drivers of network hyperexcitability, thereby offering a disease-modifying strategy to reconstruct the hippocampal E/I balance.

In summary, our findings show that early local NGF-hydrogel intervention was associated with reduced seizure burden and broad improvement in hippocampal remodeling after epilepsy. These effects involved neurogenesis-related changes, inhibitory marker preservation, attenuation of inflammatory and glial pathological changes, and improvement in synaptic and circuit-related readouts. Together, the present study suggests that the NGF-based injectable hydrogel represents a highly effective intervention for targeted neuroprotection and E/I balance reconstruction, significantly attenuating epileptic seizures.

## Data Availability

The original contributions presented in this study are included in the article. Further inquiries can be directed to the corresponding authors.

## Abbreviations

The following abbreviations are used in this manuscript:

ACSF	Artificial cerebrospinal fluid
BrdU	5-bromo-2′-deoxyuridine
Calbindin	Calbindin-D28k
CNQX	6-cyano-7-nitroquinoxaline-2,3-dione
DCX	Doublecortin
DG	Dentate Gyrus
DI	Discrimination Index
EC	Entorhinal Cortex
EEG	Electroencephalography
fEPSP	Field Excitatory Postsynaptic Potential
FS	Focal Seizure
GCD	Granule Cell Dispersion
GCL	Granule Cell Layer
GFAP	Glial Fibrillary Acidic Protein
GS	Generalized Seizure
HOPX	Homeodomain-Only Protein
IBA-1	Ionized calcium-Binding Adapter molecule 1
KA	Kainic Acid
Lamp1	Lysosomal-Associated Membrane Protein 1
Map2	Microtubule-Associated Protein 2
MEA	Multielectrode Array
NeuN	Neuronal Nuclei
NGF	Nerve Growth Factor
NOR	Novel Object Recognition
NPY	Neuropeptide Y
NSC	Neural Stem Cell
OFT	Open Field Test
PSD95	postsynaptic density protein 95
PV	parvalbumin
SE	Status Epilepticus
SGZ	Subgranular Zone
Syn1	synapsin 1
Td	tdTomato
TLE	temporal lobe epilepsy
TTX	tetrodotoxin
